# Oral Implications of Herbst Device Modification: A Case Report

**DOI:** 10.3390/children12050531

**Published:** 2025-04-22

**Authors:** Monica Macrì, Mariastella Di Carmine, Antonio Scarano, Felice Festa

**Affiliations:** Department of Innovative Technologies in Medicine & Dentistry, University “G. d’Annunzio” of ChietiPescara, 66100 Chieti, Italy

**Keywords:** Herbst, complications, anterior mandibular repositioning, device management, musculoskeletal structures adaptation

## Abstract

Background: Many studies analyse the effectiveness of the Herbst device in the treatment of dentoskeletal Class II malocclusion due to mandibular retrusion. This fixed device was devised by Emil Herbst for Class II treatment using a bite jumping, i.e., a device that holds the jaw in a forced anterior position. Comparison of the results obtained in numerous studies is difficult because they are often not comparable and not congruent due to a number of variables that prevent standardization. Methods: The purpose of the present study is to report some clinical-level considerations that may be important in order to obtain more predictable therapeutic outcomes. The simplified design of the Herbst device offers better patient comfort and easier cleanability but may show some disadvantages, such as less anchorage. Results: The device was evaluated in conjunction with the multi-bracket phase that preceded Herbst therapy and concluded after the device was removed. The therapy was performed in the absence of skeletal anchorage. Conclusions: In our opinion, standardization of therapy according to precise protocols may positively affect the therapeutic outcomes by achieving faster occlusal stabilization, more proper neuro-muscular balance, less stress on anchor units, and shorter treatment time.

## 1. Introduction

At the International Dental Congress in Berlin in 1909, Emil Herbst presented a fixed bite jump device for the treatment of class II, which held the mandible in a forced anterior position [1]. The maxilla, mandible, and muscle function changed, and the appliance was therefore considered as a fixed functional appliance [2,3]. Until 1979, there were few publications; in that year, Pancherz drew attention to the possibilities of stimulating the condylar growth of the mandible by means of this device [4,5]. The growing diffusion of this device in recent times depends on the advantage of not requiring the patient’s collaboration and the reduction in treatment times compared to other appliances, such as the twin block. The objective is to correct the sagittal discrepancy, stimulate mandibular growth, and reshape the condyle–glenoid fossa relationship, causing the anterior rotation of the mandible [6]. Therapy with the Herbst device alone typically lasts 6 to 9 months [7].

**Figure 1:**
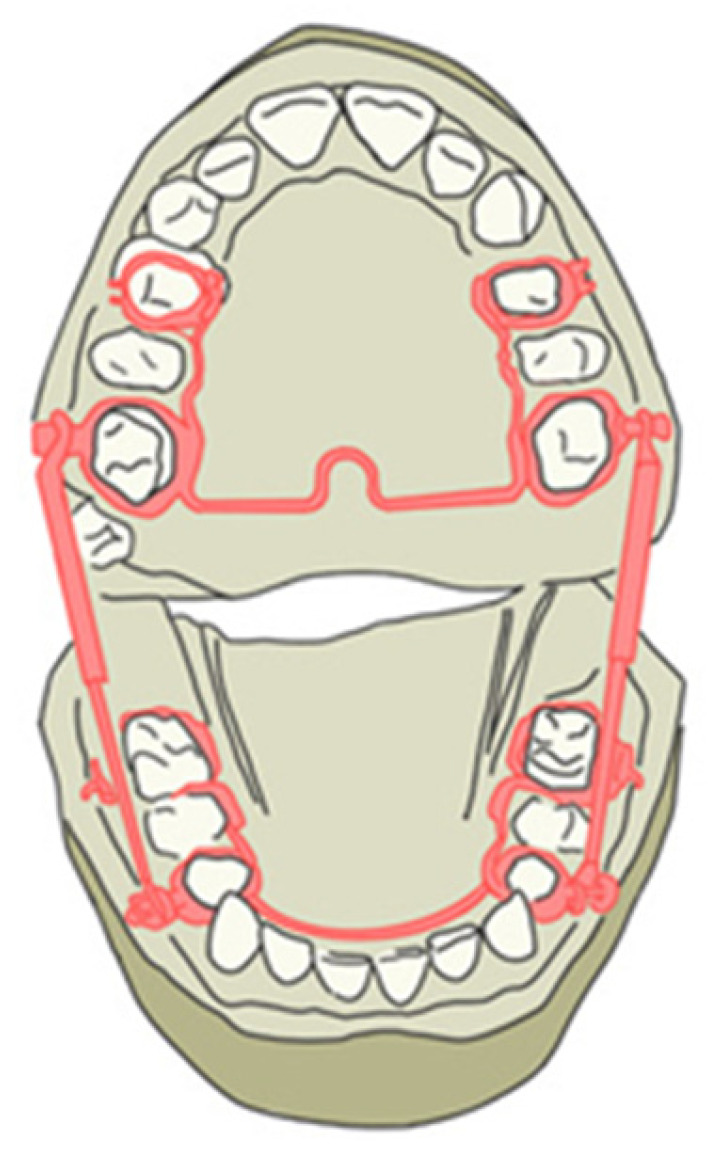
Traditional design of the Herbst appliance.

The conventional Herbst device requires the impressions of patients with permanent dentition with four bands for the permanent first molars and first premolars in each arch. The technical laboratory proceeds to solidify the bands by including the second premolar with shaped buccal and palatal bars in the anchorage unit. Therefore, the premolars and the molars of the same hemi-arch work as a single anchorage unit. The right and left units of the same arch are connected and mutually stabilized by wire elements of appropriate diameter. In the upper arch, there is a trans-palatal bar welded onto the bands of the sixths and distanced from the soft tissues to avoid mucous decubitus on the palate; in the lower arch, there is a lingual arch welded onto the first premolar, which touches the lingual surface of the lower incisors. These wire elements increase the stability of the anchoring units and reduce the effects of unwanted muscle forces (Figure 1).

The therapy can be integrated with a multi-bracket appliance (MBA), which, in some studies, represents a second phase of treatment. The modality and timing of this therapeutic phase are not specified in some studies; however, in others, it is not even foreseen, so the treatment ends only with the Herbst device [8,9]. The differences in terms of the therapeutic devices used, the sequence of their use, and the treatment length affect the outcomes related to anterior mandibular repositioning.

An anterior mandibular repositioning through devices that are either fixed or mobile should be considered a major factor. In fact, these types of appliances hold the mandible in the therapeutic position either continuously or intermittently; consequently, the adaptation of musculoskeletal structures might be affected differently. With the Herbst device, the condyles are initially repositioned inferiorly and anteriorly compared to their original position [10]. The application of the device induces an immediate movement of the condyles along the path of the articular eminence, in a different position from the initial one; this leads to potential morphological variations. However, subsequent studies indicate that at the end of treatment, there are no significant differences in the condylar position compared to the initial position [10,11]. In these studies, however, patients were treated with unspecified functional devices or devices different from the traditional Herbst device, i.e., with the use of acrylic splints.

Treatment with the Herbst device would seem not to increase the prevalence of temporo-mandibular disorders (TMDs) [12,13]. Furthermore, treatment with the Herbst device does not appear to produce clinically significant changes in the middle cranial fossa and central skull base [14,15]. Most papers available in the literature proved no association between the development of TMDs and Herbst appliance therapy [16,17]. Indeed, MRI studies demonstrated that the Herbst device did not negatively impact the function of the temporo-mandibular joint (TMJ) and, therefore, it could be applied even in patients with disc displacement who exhibited a reduction in clinical signs during therapy [18,19]. In a 32-year longitudinal long-term follow-up study, Pancherz et al. detected that the signs and symptoms of TMDs in subjects previously treated with the Herbst appliance were comparable to those of untreated adults [20]. A recent review reported no statistical evidence on the onset of TMDs following bite-jumping therapy using the Herbst appliance [21]. Moreover, this device appeared to reduce the occurrence of TMDs in treated subjects rather than exacerbate it. However, to reduce the risk of possible TMDs, especially in potentially predisposed individuals, it would be helpful to carefully evaluate the patient’s TMJ function and any TMD symptoms before treatment and to monitor the subject for the entire therapy. Therefore, an assessment of the exact position of the condylar disc in relation to the fossa with detailed three-dimensional images would be appropriate whenever mandibular advancement devices are chosen. In fact, the device is usually applied in growing patients, ensuring an adaptation of the structures from an anatomical–functional point of view. In any case, with this device, it is advisable to treat patients who have completed the dental permutation phase up to the first premolars to ensure better strategic anchorage.

Respiratory function has a huge impact not only on physiology but also affects the quality of life of patients [22]. It is known that the pharyngeal airway space is influenced by the craniofacial sagittal shape. Indeed, subjects have different airway dimensions in relation to a normal, retrognathic, or prognathic mandible [23]. The sagittal position of the mandible influences the position of the hyoid bone and tongue by the genioglossus and mylohyoid muscles, which have a close relationship with the size of the pharyngeal airway. Mandibular retrognathism is associated with a narrower airway passage. The posterior and inferior directions of mandibular growth may cause obstructive sleep apnoea syndrome [24]. In some studies, it has been observed that the Herbst appliance provides an improvement in oropharyngeal and hypopharyngeal airway dimensions [25].

To evaluate the effects and effectiveness of the Herbst device at a diagnostic level, it is necessary to identify the skeletal Class II malocclusion due to mandibular retrusion without micrognathia since the effect of the Herbst device, in any case, may only express a growth potential inherent in the individual without substantially modifying it. Therefore, a genetically small jaw cannot be substantially modified with a mandibular advancement therapy. Therefore, at the diagnostic level, it is essential to have a detailed understanding of the measurements concerning the mandibular morphology. For instance, the treatment of a Class II canine relationship greater than 7 mm associated with a protrusion of the incisors requires a partial distalisation of the upper anterior dentition, modifying the outcome of the mandibular reposition through the Herbst device beyond the effective limit achievable and compatible with the new condylar position. This can easily lead to a recurrence of the advancement treatment with the Herbst device. For this reason, a preventive preparation of the arches with an MBA is essential to correct all the problems of excessive protrusion of the anterior teeth, diastemas, alteration of the shape of the dental arches (especially even slight contraction of the maxillary dental arch since it affects the mandibular position), and asymmetries in the dental class due to particular clinical conditions, for example, rotations, dysmorphism of dental elements, etc. Secondly, a fundamental aspect for therapeutic success and for the containment of unwanted effects is the achievement of occlusal stabilization in the shortest possible time following the application of the Herbst device.

The Herbst appliance was also used in the management of severe malocclusion. For instance, Desai et al. reported a correction of Class II division 1 malocclusion characterized by a severe overjet of 13 mm thanks to the Herbst device associated with a multi-bracket appliance during peak pubertal growth [26]. Moreover, studies comparing surgical treatment to fixed functional appliances in post-adolescent borderline Class II subjects supported the predictability of fixed functional devices without the risks, costs, and post-surgery problems of the surgical approach [27]. In cases of maxillary contraction greater than 4 mm, a rapid palatal expansion should be planned before the Herbst appliance [28].

Skeletal asymmetries are detected in cases of Class II subdivision and, particularly, in some congenital craniofacial syndromes, which interfere with the symmetric growth of the mandibular body and ramal length [29]. In a recent case report, Lorenzoni et al. described the use of an asymmetrical Herbst appliance after maxillary expansion and followed by a fixed appliance to correct a mandibular Class II subdivision associated with a mandibular deficiency [30]. Regarding the management of the orofacial implications of congenital defects or systemic inflammatory diseases, a multidisciplinary approach should normally be considered. Indeed, the orthopaedic functional appliances, including the Herbst device, generally represent a part of a multi-phase therapy and could prevent a more invasive orthognathic surgery and a more complicated treatment in adults. Furthermore, the therapeutic procedures are strongly related to the variability in and severity of a patient’s congenital defect.

Concerning the asymmetries with a mandibular deviation toward the affected side in growing patients, the use of orthopaedic functional appliances with or without mandibular distraction osteogenesis has been described in the literature. However, to date, no consensus regarding the best procedure has been reached yet, probably since most of the published studies referred to case reports and reported various protocols. Moreover, more types of functional devices were also proposed to progressively treat dentoskeletal anomalies during patient growth, for instance, before, removable functional appliances, and later, the unilateral Herbst device. In less severe deformities, the asymmetric orthopaedic functional orthodontics, especially initiated at an early age and continued until skeletal maturity, was able to redirect the mandibular growth toward a favourable pattern, even avoiding the need for a future surgery [31]. In more complex cases, the functional appliances guided and stimulated the development of skeletal and soft tissues in the affected side, making the surgical interventions more predictable [32]. Indeed, functional orthodontics can intercept the unfavourable growth pattern in congenital defects, in which the asymmetry worsens with increasing age. In a juvenile idiopathic arthritis case with facial asymmetry, a digitally designed Herbst appliance combined with a rapid palatal expander was obtained by the construction bite registered with the lower jaw in an advanced and symmetric position [33]. This device, asymmetrically activated through diverse lengths of the telescopic mechanism, allowed maxillary expansion and small lateral mandibular movements, favouring the development of the hypoplastic side and the restoration of facial symmetry in eight months. Then, an 18-month straight-wire labial-fixed orthodontics corrected the overjet, overbite, and misalignment.

The Herbst device is commonly used for the management of skeletal Class II malocclusions with mandibular retrusion in growing subjects. Although this appliance is tooth-supported, many studies have confirmed a forward mandibular projection and, therefore, a skeletal modification induced by the Herbst device [34,35]. It is challenging to quantify the amount of skeletal correction since studies available in the literature differ in cephalometric variables and treatment duration. However, generally, the correction of Class II malocclusion is primarily linked to mandibular changes; indeed, maxillary growth is not restricted by the Herbst appliance. Moreover, the Herbst device allows predictable and rapid overjet reduction [36]. Therefore, skeletal Class II patients with retrusive mandibles are ideal candidates for treatment with the Herbst appliance, which is a crucial consideration when selecting a therapy [37].

An alternative approach in the management of Class II malocclusion in growing patients is removable functional orthodontics, which encompasses many types of appliances; activators, bionators, twin blocks, and Frankel appliances are commonly chosen in daily practice [38]. Both fixed and removable appliances typically represent the first treatment phase followed by a second phase of multi-bracket appliances. The functional orthopaedic phase aims to solve the sagittal discrepancy, while the fixed one manages tooth alignment, levelling, and rotations.

When a Class II malocclusion was treated early with functional appliances, the overjet and molar class relationships were corrected, reducing the progression of the initial malocclusion [39]. Ghislanzoni et al. highlighted the most notable skeletal changes with minimal dentoalveolar compensations during pubertal growth spurt [40]

Compared to removable functional appliances, the Herbst device does not require patient cooperation and, therefore, its active therapy time is shorter. Moreover, another claimed benefit of the Herbst appliance is that it maximizes the skeletal result while producing acceptable occlusal rehabilitation [41].

The present literature concerning the Herbst device mainly focuses on skeletal and dentoalveolar effects and facial profile changes. On the contrary, a small number of papers deal with the different designs of the Herbst device. Indeed, the design modification of the Herbst appliance could lead to different clinical results and determine potential side effects. Initially, during the use of traditional bands, clinicians quickly discovered that the occlusal forces were excessive and could cause band failure. Therefore, many variants of the Herbst appliance were proposed in order to improve clinical performance. For instance, cobalt–chromium alloy cast splints cemented with glass ionomer cement, bonded acrylic splints, and stainless-steel crowns were used. Nevertheless, some of those variations show disadvantages, such as difficulty in removal or a tendency for bite opening. In addition, previous studies reported different protocols of mandibular advancement (single-step vs. stepwise) and various treatment lengths, as well as a possible multi-bracket phase before the Herbst device placement to stimulate and increase sagittal mandibular correction [42,43]. Hence, every single modification could affect the final findings or duration of the orthodontic treatment. It is clear that the therapeutic results of the studies present in the literature could be influenced by the lack of standardisation of the therapeutic phases in combination with brackets and the difference in the design of the Herbst device, materials used, modes of operation, neuro-muscular adaptation, possibility of removing the device, hours of application in the oral cavity, and related prescription.

Therefore, a standardization of therapy according to precise protocols may positively influence the therapeutic result through the achievement of faster occlusal stabilization, better neuro-muscular balance, less stress on the anchoring units, and shorter treatment time.

In the present case report, we proposed a modified and simplified variant in order to obtain an appliance with simpler management.

Therefore, our study aimed to evaluate the skeletal and dental effects of a simplified Herbst device combined with a multi-bracket appliance in a young male patient.

## 2. Materials and Methods

The patient was selected among growing subjects seeking orthodontic treatment in the Department of Innovative Technologies in Medicine & Dentistry at “G. d’Annunzio” University of Chieti-Pescara. The inclusion criteria were skeletal Class II malocclusion, mandibular retrusion, normodivergence, and bilateral Class II molar and canine relationships. Patients with retained or missing teeth, with skeletal asymmetries, and individuals who had undergone a previous orthodontic therapy were excluded.

The diagnostic parameters used in selecting the patient for the current study were identified on lateral teleradiography and were as follows: ANB angle greater than 4°, SNB angle less than 78°, SN-GoMe angle between 28° and 36°, and overjet of at least 5 mm.

A young male patient met all the criteria described above. No systemic pathologies or maxillofacial disorders were detected in his medical history. The patient’s parents provided written informed consent. At the first visit (T0), extra- and intraoral photos, orthopantomography (OPT), and lateral teleradiography were taken. During X-ray examinations, the subject was positioned in the natural head position, with the teeth in centric occlusion and the lips slightly closed.

**Figure 2:**
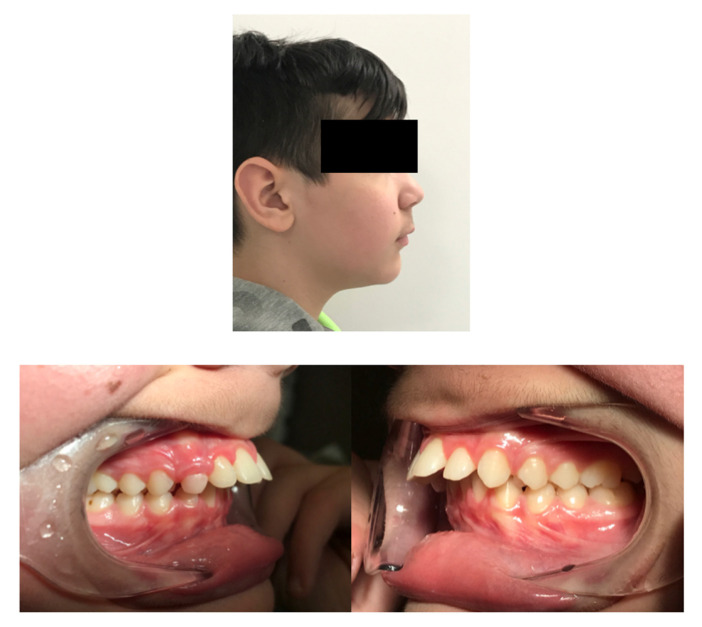
The extraoral image shows the convex profile, while the intraoral images show the Class II molar and canine relationship and the protrusion of the upper incisors with the presence of diastemas.

**Figure 3:**
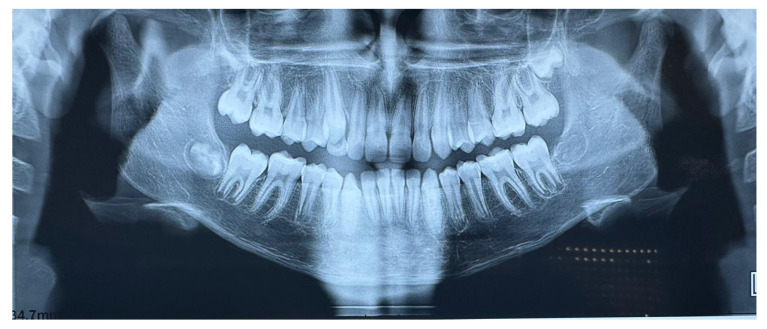
Orthopantomography at T0.

Extraoral clinical examination revealed a convex facial profile (Figure 2). Intraorally, the subject exhibited a Class II Division I type malocclusion with a bilateral Class II molar and canine relationship and an overjet of 9 mm. The maxillary incisors were proclined and, with the exception of a maxillary permanent canine, all permanent teeth were present in both arches, as shown in OPT (Figure 3).

**Figure 4:**
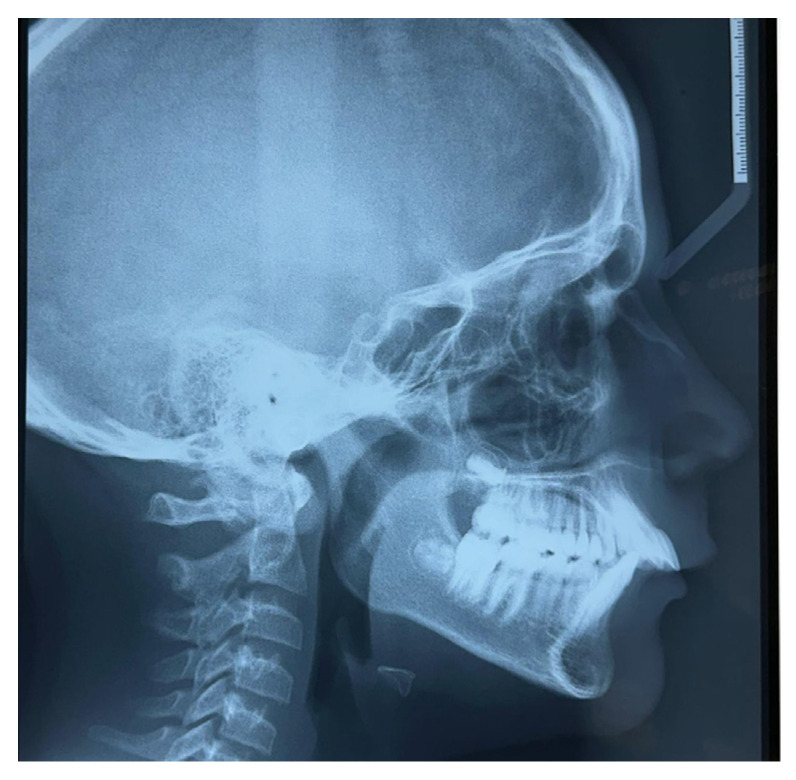
Lateral teleradiography at T0.

Lateral teleradiography confirmed the skeletal Class II Division I type malocclusion with a mandibular retrusion (Figure 4).

As mentioned above, we proposed a simplified Herbst appliance for the correction of the skeletal Class II malocclusion associated with a straight-wire labial-fixed orthodontics in order to stimulate correct mandibular growth.

### 2.1. Simplified Design of the Herbst Appliance

**Figure 5:**
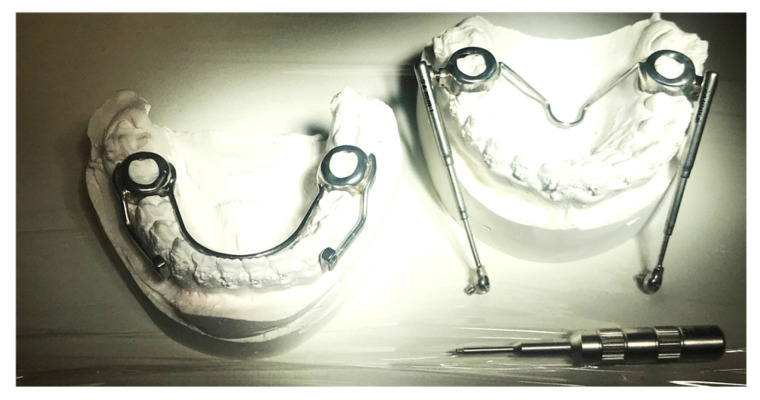
Simplified design of the Herbst appliance.

**Figure 6:**
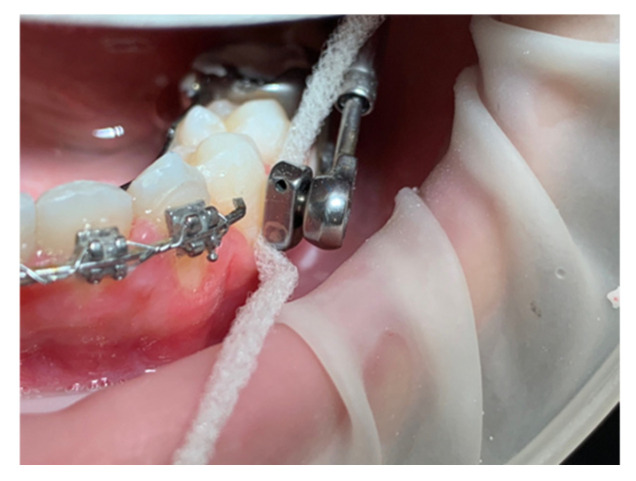
The super floss does not easily slice thanks to the space between the metal and the tooth surface.

The Herbst device can be modified by not providing the bands on the premolars. It has an arm welded onto the band of the lower molar, which extends horizontally up to the lower first premolar. The arm is spaced from the mucosa by approximately 1 mm to reduce the bulk in the oral fornix and is positioned approximately at the height of the gingival margin of the lower bicuspid. The joint of the telescopic tube is inserted at this point of the arm, which, in this version of the device, allows greater freedom in lateral movements thanks to its characteristics. The telescopic tube at the top fits onto another joint welded onto the band of the upper sixth. This allows the presence of the appliance on the upper arch to be limited to the first molars, while the rest of the upper arch is not obstructed. The design of the band provides almost total occlusal coverage to increase band retention and control over the dental effects of the applied forces (Figure 5). A space between the welded arm and the teeth must be guaranteed in order to facilitate the oral hygiene routine for the patient (Figure 6).

Table 1 summarizes the main features of conventional and simplified Herbst appliances.

### 2.2. Case Report Throughout Clinical Phases

In our clinical practice, the treatment began with a phase of alignment and levelling of the arches, elimination of the excessive proclination of the incisors, and coordination of the arches through the MBA. The coordination was checked clinically by manually guiding and asking the patient to protrude the mandible with contact of the incisal edges with approximately 2 mm of overjet.

At this point of the treatment, the simplified Herbst device was applied: the arches remained spaced apart for a period of 7 days from the application of the device in the mouth. In this adaptation period, initially only the upper incisors were in contact with the lower ones, with an overjet of 1 mm. The remaining teeth of the frontal group were supported by a rectangular sectional wire.

**Figure 7:**
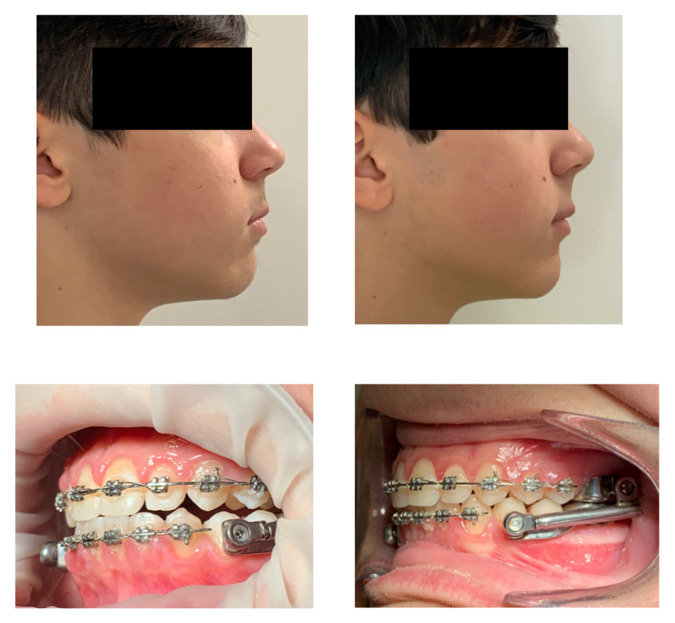
The left images refer to when the Herbst device was just cemented, after a first phase of the MBA. The right images show the modifications after 1 week.

This phase of muscular and joint adaptation was clinically visible in the patient’s face as soon as the device was cemented in the mouth and, subsequently, at the check-up after 7 days. Initially, the muscles appeared “forced” to maintain the protruded position (an aspect common to many functional devices, including the Frankel), while the muscular posture improved significantly after a week when the intercuspation occurred (Figure 7).

Through the procedure of preparing the arches with the MBA, the phase with the Herbst device, and the finalization phase with the MBA, the treatment length in this clinical case was reduced in the phase with the Herbst appliance to 5 months compared to the 6–9 months expected. This can be an interesting aspect for less permanence of the device in the mouth, greater cleanability of the oral cavity, and better patient comfort.

## 3. Results

**Figure 8:**
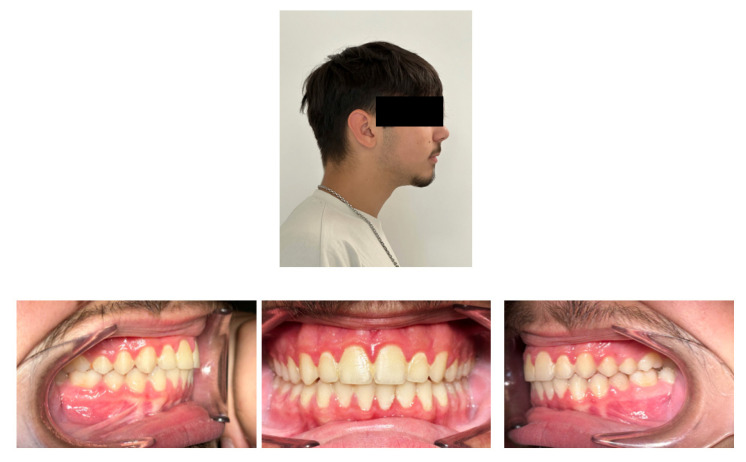
At T1, a balanced profile was achieved. The intraoral images show the proper intercuspation and dental Class I relationship.

At the end of the treatment, we obtained good results in terms of intercuspation and muscular and joint wellbeing status assessed by a clinical chart, including muscle palpation, evaluation of mouth opening, and closing dynamics. We observed an improvement in the face profile with the resolution of mandibular retrusion. The bilateral Class I molar and canine relationship and proper overjet and overbite were achieved (Figure 8).

**Figure 9:**
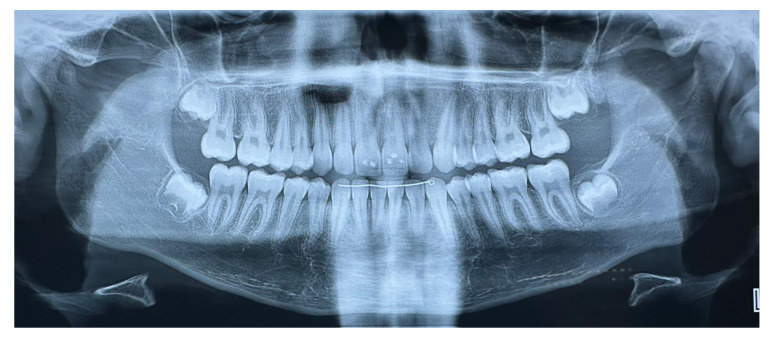
Orthopantomography at T1.

**Figure 10:**
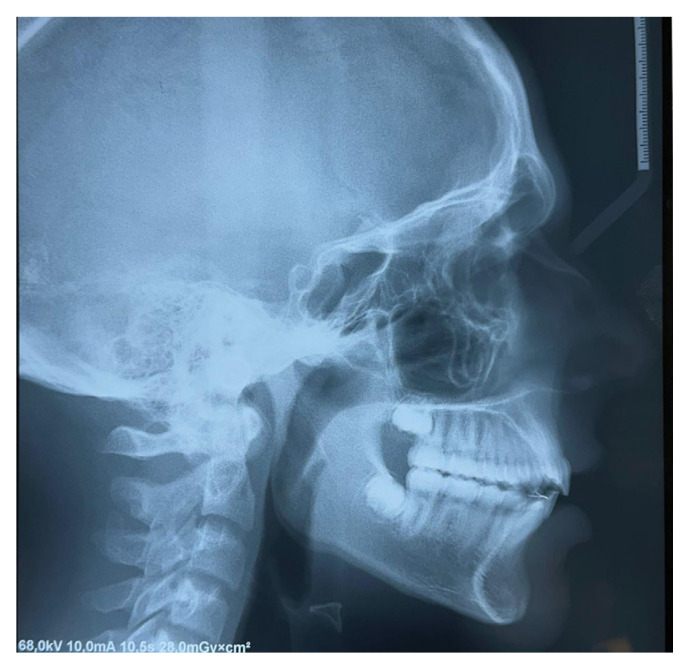
Lateral teleradiography at T1.

Orthopantomography and lateral teleradiography were taken at the end of treatment after bracket removal (T1) (Figure 9 and Figure 10). The final cephalometric analysis showed remarkable changes, including improvements in the SNA, SNB, and ANB angles, Wits appraisal, and the growth of the mandibular ramus, while the position of the lower incisors remained almost unchanged (Table 2).

## 4. Discussion

This case report described the use of a simplified Herbst device associated with an MBA for the management of Class II malocclusion in a young patient with a mandibular retrusion. At the end of treatment, both skeletal and dental improvements were achieved.

### 4.1. Clinical Observations When Using the Herbst Device

The increased overjet (compared to the conventional one that is usually brought to 0 mm) allowed for the faster achievement of intercuspation. Indeed, after a week, the overjet increased and allowed the intercuspation of the posterior teeth. In this period of time, the dentoskeletal and the neuromuscular systems underwent adaptive phenomena that allowed the dental arches to return to contact. Thanks to this modification to the classic protocol, there is the possibility of reactivating the device weeks later, when, normally, there is a mild recurrence of Class II (1–2 mm). Our goal was to obtain molar intercuspation as quickly as possible, which would be much slower with the overjet brought to 0 mm. Therefore, after approximately 1 week, the patient showed an occlusion of numerous teeth in contact in maximum intercuspation. The correct contact of the posterior teeth during the swallowing phase, which may occur after approximately 7 days, is an important aspect for receptor stimulation. When this does not happen immediately, the jaw blocked in a protruded position continues to activate the mandibular retrusive muscles during the swallowing act, with greater stress on the anchoring unit. In the aforementioned “adaptive” period, the retrusive muscular forces are very strong and are limited by the presence of a mechanical block represented by the telescopic tubes of the Herbst device: the length of these tubes influences the action on the muscles and, therefore, modifies their state of contractile activation. In this phase, the dental anchoring unit is highly stressed, considering the average load expressed in kilograms and applied over 24 h. If the dental occlusion obtainable after the adaptive phase is deficient, due to various problems, the side effects and the therapeutic result will inevitably be penalized. Therefore, to reduce stress on the anchoring unit, a first aspect to be carefully evaluated is the coordination of the arches obtained through the MBA in the preparatory phase. If the arches are not well coordinated, during the mandibular advancement phase using the Herbst device, it will not be possible to obtain stabilization in the desired position since the dental interference in the occlusion could cause a distalizing recurrence on the mandible.

### 4.2. Clinical Observations After Using the Simplified Design

Concerning the need for rapid occlusal stabilization in order not to stress the dental anchoring elements, avoiding both excessive loads and unwanted movements (for example, lower dental protrusion), we proposed the use of occlusal covering bands. This band typology occupies the occlusal surface: since the occlusion expected after device cementation is a dental Class I relationship, the bands of the upper and lower molars are in mutual contact, which determines a significant occlusal rise that is at least 2 mm, considering the metal thickness. Hence, in the stabilization phase, only the permanent first molars occlude, while the remaining teeth in the arch are not in mutual contact. The spontaneous eruption of the teeth is fundamental in order to obtain a distributed occlusal load; this can require months and may not necessarily be completed. The dental elements that occlude more easily are the second molars that cannot remain united with strings during this phase. In fact, at the level of the second molars, the arch cannot be used due to the presence of welding on the molar bands, while the other teeth are united with a rectangular wire in order to avoid unwanted movements. After 6–9 months of the Herbst device, according to the traditional times recommended in the literature, the first molars will not be in occlusion since other dental elements will be extruded. This effect can be dangerous due to uncontrolled movements, not only on the vertical plane but also on other spatial planes. The teeth that have not been stabilized will be able to vestibularize or lingualize as well as extrude. In fact, after device removal, we usually notice the spontaneous and unwanted eruption of the second molars that cannot be retained through the use of rectangular wire sectionals, different from the anterior sector. The precontact of these teeth favours the occlusal interference and posterior sliding of the mandible, as the stomatognathic neuromuscular system seeks stabilization. A further aspect is the occlusal flat design of this band type. This design prevents intercuspation between the molars when they are in contact, a factor which is indispensable for occlusal stabilization. In fact, the narrow occlusal intercuspation allows the effective stabilization of mandibular anterior repositioning using the Herbst device. Therefore, it is reasonable to think that in the initial stages, the anchoring teeth are predominantly subjected to the forces of mandibular retrusion. In fact, during a potential temporary removal of the telescopic tubes (for example, due to leakage following an excessive opening of the mouth, as in the historical models of this device, or due to the unscrewing of the lateral fixing screw), a spontaneous retrusion of the mandible may occur, which confirms the presence of strong retrusive muscle forces during the first months of therapy. To overcome this drawback, we used the Herbst device with partial occlusal coverage bands.

This simplified Herbst device does not include bands on the premolars; instead, it has an arm welded onto the band of the lower molar, which extends horizontally up to the first lower premolar. This arm is spaced from the mucosa by approximately 1 mm to reduce the bulk in the oral fornix and is positioned approximately at the height of the gingival margin of the first premolar, thus including only the first molars in the upper arch. The intention is certainly to promote home hygiene and reduce the incidence of tooth decay on the anchoring teeth. These variations reduce the bulk at the premolar level since the metal structure is eliminated. We have found that in the upper arch, the retrusive and occlusal muscular forces determine stress only on the sixths, which are forced to bear the load. These forces could cause a selective intrusion of these teeth in the upper arch with consequent occlusal variation and decubitus of the trans-palatal Goshgarian bar. This inconvenience may require appliance removal with consequent loss of chair time and loss of the therapeutic position reached at that moment. We have overcome this inconvenience by applying a greater distance of the bar from the palatal mucosa during the manufacturing phase, but always maintaining monitoring of the situation during clinical checks.

In the lower arch, only the first molars suffer the mesializing forces instead of the four teeth per arch in the original device design. The spatial instability of the metal elements can also occur on the lower arm welded to the band of the lower molar. The muscular force may determine the creation of a lever arm, which discharges its force on the lower molars; possible slight displacements of the terminal part of the arm could create a direct mucosal decubitus or an unwanted vestibularization of the first premolar, which cannot be controlled by wire sectionals. The reduced space between the arm and the dental surfaces of the premolars allows the rectangular wire section used in the anterior sector from canine to canine to reduce the displacement of the dental elements. Therefore, the lower premolars remain without any movement control. The premolars may vestibularize and come into contact with the appliance. Therefore, we recommend increasing the space between the welded arm and the teeth. Moreover, the patient will be able to monitor the status of the device during daily home oral hygiene procedures. In fact, the area can be cleaned with a specific type of dental floss.

An exact comparison with previous research was complicated since each study presented a different type and/or protocol of the Herbst device. Karbach et al. underlined the multiple modifications of the Herbst appliance used in daily orthodontic practice in relation to patients’ malocclusions; therefore, the possible variants of this device could actually be underestimated [44]. For instance, Metzner et al. observed a greater predictability of a modified Herbst appliance during a mesialization of lower molars with lingual bracket orthodontics than a conventional Herbst device [45]. On the contrary, Marchi et al. detected no substantial differences between crown and acrylic splint Herbst appliances [46]. However, despite this methodological heterogeneity, the studies available in the literature demonstrated the correction of Class II malocclusion, especially in cases with mandibular retrusion, thanks to fixed functional devices.

In our research, we used cephalometric measurements to analyse the sagittal changes in jaws. Different cephalometric variables and various treatment lengths have been proposed in the literature, impeding precise comparison [47]. However, generally, the available studies on the Herbst device agree on the effectiveness in the mandibular advancement associated with an improvement in patients’ profiles.

Regarding maxillary changes, we observed no substantial restriction of maxillary growth; indeed, SNA decreased by 0.5° at the end of therapy. This outcome aligned with previous studies [2,48]. The maxillary modification is a result of mandibular projection, which modifies the condylar/fossa relationship.

Our results revealed a positive mandibular response to orthopaedic input and, hence, corroborated those obtained by Marchi et al., who confirmed an improvement in the sagittal relationship between jaws after a modified Herbst device combined with a fixed appliance [49]. SNB increased by 4.5°, unlike in earlier studies reported lower values [50]. ANB decreased by 2.5°, similar to Marchi et al. [46]. In contrast, Farouk et al. detected a lower increase in ANB, although their treatment duration was longer than ours [51]. Marchi et al. observed similar improvement in ANB in two different types of the Herbst appliance and confirmed skeletal changes [46]. In contrast, Zymperdikas et al. noted that the effectiveness in correcting Class II malocclusion was related mainly to dentoalveolar rather than skeletal effects [52].

The slight increase in lower face height with a clockwise rotation of the mandibular plane was consistent with previous results [46,53]. Higher facial height is related to the geometric effect of mandibular advancement [46]. Similar to Sangalli et al., we found more substantial changes in the sagittal values than in the vertical ones [54].

We noticed a 6.5 mm reduction in overjet as well as a molar relationship correction at the end of treatment. The decrease in overjet was similar to that obtained by Wigal et al. [35]. However, in the literature, the overjet reduction ranged from 3.3 to 9.8 mm [55,56].

In contrast to other research, we observed a good control of lower incisor proclination, likely due to the support of a rectangular sectional wire [57]. The upper incisors exhibited a backwards movement, as reported by Tomblyn et al. [57]. The improvements in the overjet and molar relationship were linked to both skeletal and dental changes, as confirmed by Tomblyn et al. [57]. Moreover, these authors observed the stability of skeletal changes after a multi-bracket appliance. We also found an overbite reduction with molar and lower incisor intrusion, as confirmed by previous studies [57,58].

The simplified Herbst appliance enhanced the occlusal relationships, corroborating previous works [59]. Therefore, every single skeletal and dental modification contributed to the overall final result. Hence, the Herbst appliance and its modifications promoted mandibular growth and reposition and, consequently, improved the occlusal relationship and facial aesthetics.

We proposed a simplified Herbst device, where the appliance remained in the patient’s mouth for a limited period of time (five months); indeed, for a longer period, a reinforced Herbst device should be requested in order to avoid possible breakages [41]. Moreover, the short treatment length minimizes the onset of irritation and sores on the oral mucosa. The Herbst device proposed in our study had no premolar bands, thus reducing the risk of caries and root resorptions on the bicuspids.

Functional orthodontics is a widely accepted therapy choice for managing Class II malocclusion and comprises both removable and fixed devices. In the literature, different types of functional appliances have been proposed, but none of them seems to show more effectiveness in the correction of Class II malocclusion. A recent paper comparing the twin-block appliance with the Herbst one demonstrated the efficacy of both devices, with similar dentoskeletal changes in malocclusion correction [60]. However, the Herbst device increased the length of the mandibular body, while the twin-block one improved the facial profile. Therefore, the authors concluded that the choice of appliance type depended on the treatment benefit related to the initial patient characteristics. Recently, a mandibular advancement with clear aligners was proposed in order to position the mandible forward simultaneously with the dental alignment. Wu et al. compared the clear aligners with mandibular advancement with the traditional functional appliances [61]. The authors concluded that both clear aligners with mandibular advancement and removable and fixed functional devices corrected the Class II malocclusion; however, the clear aligners with mandibular advancement showed more dentoalveolar effects, whereas the twin-block appliance and, particularly, the Herbst appliance showed more skeletal effects. Another paper investigating both clear aligners with mandibular advancement and the Herbst appliance reported a better control of lower incisor inclination with clear aligners [62].

The primary drawback of removable functional appliances is correlated with the patient’s cooperation; in fact, treatment discontinuation rates may vary from 9 to 15% [63]. Compared to other functional appliances, the Herbst one offers therapeutic benefits, i.e., continuous daily therapy, shorter treatment duration, and no speech interference [64]. Moreover, taking advantage of residual growth, the Herbst device is also indicated in late adolescents.

In addition to the primary objective of redirecting mandibular growth, functional appliances may determine an improvement in airway patency. The development of the dentofacial complex is closely linked to that of the pharyngeal airway; indeed, in patients with a retrognathic mandible, the posterior tongue position reduces the airway volume. Tahmasbi et al. reported an augmentation in airway size after the twin-block appliance, whereas no changes were observed following the Seifi device; however, the intermaxillary relationship was enhanced with both types of appliances [65]. Oliveira et al., using CBCT scans, detected a significant increase in oropharyngeal airway calibre in growing subjects with a mandibular retrusion after the Herbst device [66].

We limited our findings to 2D data of the orthopantomography and lateral teleradiography. We did not analyse the condyle–glenoid fossa complex three-dimensionally. CBCT would be useful in investigating possible changes regarding the condylar position and volume following the placement of the modified Herbst device. A 3D analysis could accurately evaluate the possible modifications in oropharynx volume. Moreover, we did not analyse whether our results remained stable in the long term.

Thus, 3D modifications concerning dentoskeletal patterns and the upper airway on a larger study sample using this simplified Herbst device could be the subject of further investigations.

The current simplified Herbst appliance associated with a multi-bracket therapy was able to guarantee results similar to those described in previous studies. Indeed, once the congruence of the arches was achieved, thanks to its simple design, the Herbst device proposed in our study postured the mandible forward in a shorter treatment duration without any discomfort for the patient.

## 5. Conclusions

Within the limitations of a case report, we found a normalization of the dentoskeletal patterns and an improvement in the facial profile. We observed a mandibular advancement in a shorter treatment duration associated with good control of lower incisor proclination. A multi-bracket orthodontics prior to the positioning of the simplified Herbst appliance created an appropriate coordination of the arches, which allowed us to reduce the duration of the Herbst appliance and prevent any unwanted effects related to this device. Occlusal stabilization is fundamental for the acquisition of a new neuromuscular engram, which limits the deleterious effects due to the action of the muscles on the anchoring units. Moreover, thanks to its simplified design, the current device was well tolerated by the patient, whose compliance was not requested.

Therefore, the modified Herbst appliance provides an aid for clinicians in the correction of Class II malocclusions with retrusive mandibles in a shorter therapy duration. The changes made in the current Herbst appliance are particularly indicated for subjects with a high risk of caries, for patients with poor oral hygiene, and for patients who do not tolerate telescoping arms in their mouth for an extended period.

Further research should focus on simpler device designs and innovative materials to reduce the treatment time of the Herbst appliance. Moreover, future studies should be conducted using standardized protocols and longer follow-up periods.

## Figures and Tables

**Table 1:** Main differences between traditional and simplified designs.

	Traditional Design	Simplified Design
Total number of bands per arch	4	2
First molar bands	Normal	Partial occlusal coverage
Goshgarin trans-palatal bar	Present	Present
Welded lingual arch	Present	Present
Welded arm extension from molar to first premolar	Present	Absent
Left and right telescopic tube	Present	Present

**Table 2:** Pre- and post-treatment cephalometric values.

	Reference Values	T0 Measurements	T1 Measurements
SNA angle	82°	82.5°	82°
SNB angle	80°	75°	79.5°
ANB angle	2°	5°	2.5°
Wits appraisal	0 mm	4 mm	2 mm
SN-GoMe angle	32°	35°	36°
SN-SnaSnp angle	8°	7°	6°
SnaSnp-GoMe angle	26°	28°	30°
*p*.O.-GoMe angle	16°	19.5°	17°
GoMe-GoPc angle	126°	128°	122°
GoMe-GoN angle	73°	71°	73°
GoN-GoPc angle	53°	57°	49°
SGn-SN angle	67°	68°	69°
SGo/NMe	62%	62%	63%
I+/I− angle	131°	106°	130°
SnaSnp-I+ angle	109°	126°	105°
GoMe-I− angle	90°	102°	102°
Overjet	2 mm	9 mm	2.5 mm
NB-LsPgC angle	8°	17°	11.5°

## Data Availability

The original contributions presented in this study are included in the article. Further inquiries can be directed to the corresponding author.
